# Assessment of aqueous graphene as a cancer therapeutics delivery system

**DOI:** 10.1038/s41598-025-98406-0

**Published:** 2025-05-02

**Authors:** Amanpreet Kaur, Eleftheria Babaliari, Victor M. Bolanos-Garcia, Mary Kefalogianni, Sotiris Psilodimitrakopoulos, Paraskevi Kavatzikidou, Anthi Ranella, Morteza Ghorbani, Emmanuel Stratakis, Dmitry G. Eskin, Iakovos Tzanakis

**Affiliations:** 1 Faculty of Health, Science and Technology, Oxford Brookes University, Headington, Oxford, OX3 0BP UK; 2 Foundation for Research and Technology - Hellas (F.O.R.T.H.), Institute of Electronic Structure and Laser (I.E.S.L.), Vasilika Vouton, Heraklion, 70013 Greece; 3 Department of Biological and Medical Sciences, Faculty of Health and Life Sciences, Oxford Brookes University, Gipsy Lane, Headington, Oxford, OX3 0BP UK; 4 Department of Physics, University of Crete, Heraklion, 70013 Greece; 5 Sabanci University Nanotechnology Research and Application Center, Tuzla, Istanbul, 34956 Turkey; 6 Brunel Centre for Advanced Solidification Technology, Brunel University London, Kingston Lane, London, UB8 3PH UK; 7 Department of Materials, University of Oxford, Parks Road, Oxford, OX1 3PH UK; 8 Oxford Target Therapeutics Ltd., OX3 0BP, Oxford, UK

**Keywords:** Aqueous graphene, Ultrasonic liquid phase exfoliation, Cytotoxicity, Cancer treatment, Targeted drug delivery, Engineering, Materials science, Nanoscience and technology

## Abstract

Graphene is a nanomaterial used in health and oncology settings. However, several reports have raised the alarm about potential toxicity. This study addressed this concern and determined the in vitro cytotoxicity of few-layer graphene (FLG) flakes produced in bespoke ultrasonic reactors using benign methods. The use of graphene flakes as a potential sensitising agent and a carrier for drug delivery in cancer cells was evaluated. To this end, aqueous based FLG suspensions were systematically characterised using UV-Vis, Raman spectroscopy and High-resolution Transmission electron microscopy (HR-TEM). Cell toxicity characterisation (e.g., cell viability assays using 3-(4, 5-dimethylthiazol-2-yl)-2, 5-diphenyltetrazolium bromide (MTT) and cell membrane integrity) of FLG in water were performed together with charge coupled device (CCD) and second harmonic generation (SHG) imaging of live cells in graphene solutions. Collectively, our findings show that NIH 3T3 mouse fibroblast and human fibroblast cells survival was higher than 80% and 90%, respectively upon treatment with the FLG fraction (~ 16 µg/ml ) recovered after centrifugation at 2000 revolutions per minute (RPM). In contrast, the cervical cancer cell line HeLa exposed to similar concentrations of FLG flakes resulted in approximately 30% cell death arguing in favour of a sensitising effect in cervical cancer cells.

## Introduction

The graphene family of nanomaterials comprises single-layer graphene (SLG), few-layer graphene (FLG), graphene oxide (GO), 3D-graphene scaffolds and reduced graphene oxide (rGO). All these graphene types are the subject of intense investigations to establish their potential applications in biomedicine and biotechnology^1^. The ability to functionalize the graphene surface represents a promising method for developing drug delivery systems with precise control of drug release^2^. For their clinical use, drug-containing nanomaterials must comply with high standards of quality, safety and efficacy. This includes quantification of cell toxicity in vitro and in vivo^3^.

In this regard, many research efforts have been concentrated on assessing the toxicity of a wide range of graphene-based nanomaterials (GBN), from chemically functionalized graphene to graphene dispersed in harsh solvents such as N-methyl-2-pyrrolidone (NMP), dimethylformamide (DMF) or in surfactants such as Sodium Dodecylbenzene Sulfonate Surfactant (SDBS) and Sodium Cholate (SC), potentially causing damage to the cellular membrane and subcellular components^4^. Moreover, graphene derivatives such as GO, rGO and liquid-phase exfoliated (LPE)-graphene treated in organic solvents or surfactants have been shown to be cytotoxic^4,5^. Residual contaminants may add to the risk of graphene-induced toxicity in cells^1^. Moreover, various studies have been conducted on different forms of graphene to observe their effects on cell toxicity^4^. Such studies have revealed that the number of layers, lateral size, purity, surface chemistry and hydrophilicity of graphene flakes influence cell toxicity^6^.

An early in vitro study reported that GO was the most cytotoxic, causing damage to mitochondrial and plasma membranes^7^. A more recent study by Ruiz and collaborators (2020) noted that the toxic response to graphene in vitro and in vivo was dependent on features of its surface introduced during manufacturing processes. These findings are in agreement with a study reporting^8^the effects of UV-irradiated GO in human breast cancer cells (MCF-7), which induced the production of Reactive Oxygen Species (ROS), resulting in cancer cells death. Moreover, an independent study showed that treating MCF-7 cells with MXene exfoliated flakes and UV radiation synergistically increased cytotoxicity, ultimately resulting in apoptosis^9^.

In contrast to GO, FLG dispersions in aqueous environments produced with top-down approaches such as liquid exfoliation are good candidates for biological applications because they are free of chemicals compared to their counterparts prepared in toxic reagents^3^. Additional advantages of aqueous based FLG include reduced transportation costs and low health and safety risks, and the possibility of tailoring formulations to satisfy end user needs. As 2D materials-based wearable electronics and implantable sensors interact with organs and tissues (e.g., skin, brain, mouth, arteries), so the development of a safe and “sustainable-by-design” (SSbd) concept is critical to avoid the use of gallons of toxic organic solvents for their synthesis. Therefore, to achieve this goal, the Graphene flagship project (2013–2023) addressed green chemistry technologies for the sustainable manufacturing of 2D nanomaterials^10^.

It has been reported that the lateral size and thickness distribution of graphene nanosheets are important contributors to cell toxicity^11^. Recent studies^10^ found that three materials, namely GO (average lateral size: 1.18 μm), FLG (average lateral size: 300 nm), and smaller FLG sheets (lateral size: 36 nm), were cytotoxic (more than 90% cell death) towards normal human bronchial epithelial (NHBE) cells at concentrations as low as 5 µg/mL. Błażej Scheibe et al.^12^ investigated biological effects induced by TiC (from family of other 2D nanomaterials) on the human fibroblasts (MSU1.1) and their analyses revealed that exposure to higher concentrations (≥ 400 µg/mL) of TiC, Ti2 AlC, and Ti3 AlC2 particles with the sizes < 44 μm could be toxic, inducing a significant cytotoxic effect via oxidative and mechanical stress generation which was linked to strong mechanical and chemical cell membrane disruption. Ganguly et al.^13^ revealed green reduced RGO-filled semi-IPN hydrogels are non-cytotoxic towards MG 63 cell-lines without/negligible cell death in live-dead assays. According to their study, the electric pulse release experiments exhibited similarities to the pH-responsive drug release profiles, where the electric pulse functioned as an “on-off” stimulant in the drug release behaviour analogous to that of variable medium pH.

In previous studies we demonstrated the importance of ultrasonic processing (USP) parameters and frequencies, either low frequency (range of 20 kHz) or the combination of low (kHz) and high frequency (range of 1 MHz), to produce high-quality FLG flakes in eco-friendly solvents with desired size and thickness^14–18^. We have also revealed the applicability of USP to produce high quality FLG in pure water^19,20^.

Building on this knowledge, in this study we investigated whether our FLG dispersions meet the biocompatibility requirements for use as a potential biomaterial and drug carrier. To this end, graphene was exfoliated in pure water using a novel, energy-efficient, effective, facile and scalable ultrasonic liquid-phase exfoliation (ULPE) technique. We also investigated the effect of the FLG dispersions thus produced on cell viability in healthy and cancer cells, as a first step to establish the true potential of this graphene product for drug delivery to cancer cells. Conventional “off-tumour” cancer treatment approaches were associated with the non-specific distribution of chemotherapeutic drugs throughout the body via blood circulation, which risked causing damage to healthy tissues such as nausea, hair loss, fatigue, decreased resistance to infection, infertility, and organ damage while attempting to destroy malignant cells. Due to the off-target effects of conventional chemotherapeutic drugs, clinicians reduce the drug dose required to eradicate all cancer cells, thereby compromising therapeutic efficacy. Our study showed that graphene was non-cytotoxic to fibroblasts but exhibited moderate cytotoxic to cervix cancer (HeLa) cells, suggesting that bio-friendly G-nanocarriers prepared in aqueous and biological media might have great potential for their use as drug carriers and the sensitisation of cancer cells.

## Materials and methods

Graphite powder (GP) (Alfa Aesar 300 mesh, maximum 56 μm) and ultra-pure de-ionized water (DIW) (Hexeal Chemicals, UK) were used as starting materials for exfoliation. Silicon wafers (orientation < 100 > from Pi-Kem, UK) cleaned with isopropanol (99.9%, Merck Life Sciences, UK) and acetone (99.9%, Merck Life Sciences, UK), and holey carbon coated copper grids (300 mesh, Agar Scientific, UK) were used for examination of FLG flakes.

### Graphene exfoliation

ULPE of graphite was performed in DIW according to our established protocols^17–20^. The initial graphite concentration was 0.4 mg/ml and the solution temperature was maintained at 40 ± 1^o^C during continuous sonication of 2 h using a Heilscher UP400St piezo-electric transducer with a titanium sonotrode (tip diameter of 22 mm) operating at a frequency of 24 kHz with a peak to peak amplitude of 23 μm and integrated with a chiller unit (Cole Parmer Stuart SRC5). Detailed experimental procedures can be found in our previously published work^17,20^. Immediately after 2 h of ULPE, dark graphene poly-dispersions were pipetted and centrifuged at three different centrifugation speeds, 500, 1000 and 2000 RPM for 30 min using a SciSpin One Benchtop centrifuge to obtain approx. 75 ml of supernatants of varying concentrations 40 µg/ml, 30 µg/ml and 16 µg/ml respectively.

### Graphene characterisation

UV-Vis absorption spectra of fresh supernatants were recorded in the wavelength range of 200–800 nm with a Cary-60 spectrophotometer (Agilent Technologies) using quartz cuvettes (volume 3.5 ml, an optical path length of 10 mm, Agilent Technologies). A dual-beam mode and baseline correction were used throughout the measurements to scan the samples. Further, the examined supernatants were drop-cast onto a cleaned silicon wafer substrate and subsequently dried in a vacuum oven prior to Raman investigations. Micro-Raman analyses of the drop-cast samples were performed using a Horiba LabRAM HR Evolution confocal Raman spectrometer with 532 nm laser excitation. Data collection was performed for 20–30 random registered graphene flakes in the range from 1200 to 3100 cm^−1^ using a 100× objective, averaging 10 s acquisition, and using automatic cosmic rays removal. Simultaneously, 2–3 drops were put onto a holey carbon coated copper grid placed on a filter paper to wick away excess solvent and were dried completely for transmission electron microscopy (TEM) investigations. TEM/High-resolution (HR-TEM) analyses were performed to investigate the individual flakes using a JEOL 2100 F field emission gun microscope operating at 200 kV.

### Graphene flakes solution preparation: Dilution with phosphate buffer saline (PBS)

Three solution groups of graphene at varying concentrations in DIW (i) 500 RPM (40 µg/ml) (ii) 1000 RPM (30 µg/ml) and (iii) 2000 RPM (16 µg/ml) were used. By incubating adherent fibroblasts with the above solutions and serial dilutions of them, the cells were swollen and burst *(data not shown)*. Various isotonic solutions were used, e.g. phosphate buffered saline (PBS) (pH 7.4, water-based salt solution, sterile-filtered, suitable for cell culture, purchased from Sigma Aldrich) in different concentrations (1x and 2x) and Dulbecco’s modified Eagle’s medium (DMEM-high glucose (HG) with and without (wo) foetal bovine serum (FBS)). Three concentrations of aqueous graphene flake dispersions (40 µg/ml, 30 µg/ml, and 16 µg/ml) were used to produce two distinct solution groups. (a) PBS (2x) at 1:2 dilution, and (b) DMEM_HG without FBS/Penicillin-Streptomycin (PS) at 1:2 dilution, both in 15 ml falcon tubes (Sarstedt, Germany). All the tube falcons were bath sonicated using ultrasonic system (Elmasonic S30H) (ultrasonic frequency 37 kHz, ultrasonic power 80 W, maximum peak power 320 W) for 1 h at room temperature (20^o^C) prior to their incubation with cells.

### Cell seeding

*NIH 3 T3 mouse fibroblast cell seeding*: NIH 3 T3 mouse fibroblast cells were grown in cell culture flasks using DMEM-HG (4500 mg/L glucose) (Gibco™, USA) supplemented with 10% FBS (Gibco^™^, USA) and 1% PS (Gibco^™^, USA) at 37^o^C in a 5% CO_2_ incubator, with medium renewal every 3–4 days. NIH 3 T3 fibroblasts were seeded in concentration of 50000 cells/ml in DMEM in 48-well plates (Corning Incorporated, USA) and cultured for 24 h in order to reach confluency 80–90%. After 24 h, the cells were washed twice with 300 µl PBS (1x) (Sigma Aldrich^®^, Germany), a buffer solution of pH = 7.4, to remove any cell debris. After the washing step, the cells were incubated with 500 µl of the different graphene concentration groups (40 µg/ml, 30 µg/ml, 16 µg/ml) (1:2 (50%) and 1:1(100%) serial dilutions in DMEM-HG.

*Human fibroblasts (Retinal pigment Epithelium-Cells, RPE) and cervical cancer cell line HeLa cells seeding*: Human fibroblasts and HeLa cells were grown in cell culture flasks using (DMEM)–HG (4.5 mg/ml glucose) (Gibco™, USA) supplemented with 10% FBS (GibcoTM, USA) and 1% PS (GibcoTM, USA) at 37^o^C in a 5% CO_2_ incubator, with medium renewal every 3–4 days. For these experiments, graphene dispersions were produced in PBS using similar ULPE experimental conditions as discussed in 2.1. The rationale behind choosing PBS as an exfoliating media, instead of water was to prevent multiple dilutions, which was the case in using water (see Sect. 2.3) and reduce processing steps.

### Cytotoxicity assays

The MTT assay was used to measure cellular metabolic activity as an indicator of cell viability, proliferation and cytotoxicity. It is a colorimetric assay based on the reduction of yellow tetrazolium salt (MTT) (Sigma Aldrich^®^, Germany) to its insoluble formazan, which had a purple colour. 5 µl of MTT solution (5 mg/ml in PBS) was incubated for 4 h at 37°С. Finally, the MTT supernatant was discarded and the insoluble purple formazan crystals produced by live cells were dissolved in 500 µl of DMSO (dimethyl sulfoxide) (Sigma Aldrich^®^, Germany). The plate was left at 4 ^o^C, and then all the solutions (at 100 µl per well) were transferred to a 96-well plate (Corning Incorporated, USA) and this plate was read at the ELISA microplate reader (optical density at absorbance 545 nm). The raw data was saved in.txt format and normalized towards the mean of control group (only cells with in serum free DMEM) as shown in Eq. (1):

1 $$Normalised \;Cell\; viability (\%) = (A \;sample)/(Mean \;A \;control) \times 100\%$$

The data were expressed as mean ± standard deviation (SD) in relation to the control. The data were subjected to one-way ANOVA followed by post hoc Tukey HSD for multiple comparisons between pairs of means. Statistically significant difference between experimental results was indicated by *p* < 0.05.

### In vitro study of cell morphology via live imaging experiments

50,000 NIH 3 T3 cells/ml were cultured on the PET flat substrates. The three different graphene solution groups as previously identified in Sect. 2.1 of 500 RPM (40 µg/ml), 1000 RPM (30 µg/ml) and 2000 RPM (16 µg/ml) were used for the cell culture experiments. Each group was diluted in DMEM-HG wo FBS wo PS (1:2) and ultrasonicated for an hour. Afterward, 1 ml of each graphene solution was added to each PET flat substrate and left undisturbed for 24 h. After 48 h of culture, the samples were removed from the incubator and washed with PBS. Afterward, they incubated with the fluorescent dye Biotracker 650 Red Nuclear Dye (1:1000 in DMEM-HG wo FBS wo PS) for 1 h at 37^o^C. Subsequently, they were washed with fresh DMEM-HG wo FBS wo PS to eliminate any excess dye. The samples were then transferred to glass-bottomed petri dishes (Thermo Scientific, OH, USA) and seen under a custom-built multiphoton microscope at 20× magnification.

**Fig. 1:**
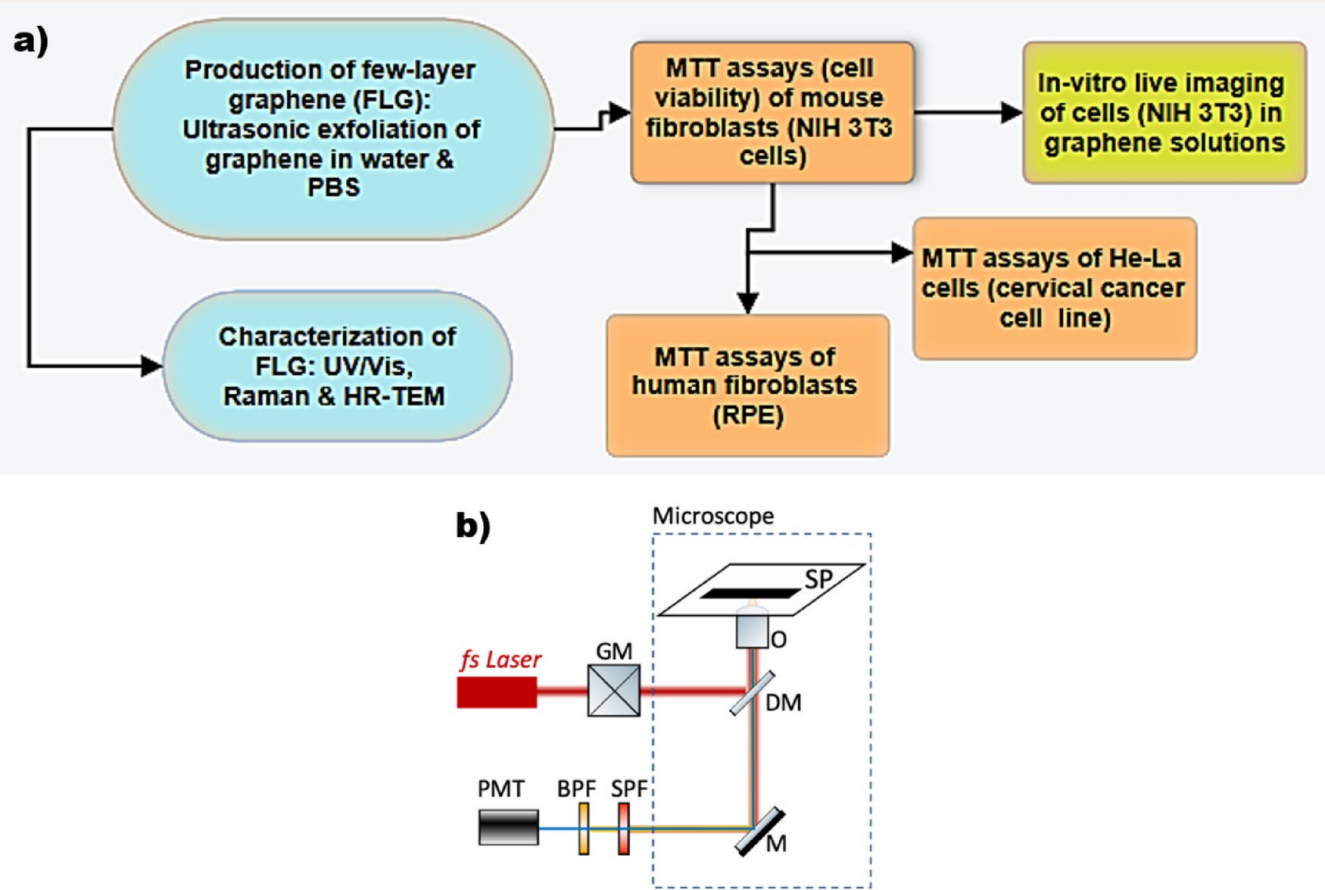
(**a**) Flowchart representing experimental processes; (**b**) Schematic representation of the experimental setup used for simultaneous 2p-F and SHG imaging microscopy of Biotracker 650 Red Nuclear dye and graphene, respectively. Abbreviations: GM, galvanometric mirrors; DM, dichroic mirror that reflects wavelengths longer than 700 nm and lets pass wavelengths shorter than 700 nm; OL, objective lens; SP, sample plane; SPF, short-pass filter that lets pass wavelengths shorter than 680 nm; BPF, band pass filter that lets pass wavelengths in the range 620 ± 26 nm or band pass filter that lets pass wavelengths in the range 514 ± 1.5 nm; PMT, photomultiplier tube.

To evaluate changes in cell morphology as a function of different concentrations of graphene solutions, NIH 3 T3 cells were imaged using multiphoton microscopy. By using a single wavelength fs laser source, we excite simultaneously two-photon fluorescence (2p-F) from the Biotracker 650 Red Nuclear Dye in the nucleus of live cells and SHG from graphene. Figure 1a depicts a flowchart that explains the experimental design of this work. Our custom-built multiphoton microscope (Fig. 1b) is based on a 1030 nm fs laser (Pharos-SP, Light Conversion, Vilnius, Lithuania), which is passing through a pair of galvanometric mirrors (6215 H, Cambridge Technology, Bedford, MA, USA) before entering into an inverted microscope (Axio Observer Z1, Carl Zeiss, Jena, Germany)^21,22^. The use of this single wavelength (i.e. 1030 nm) laser overcomes the need of using an excitation filter. The beam is then reflected by a short pass dichroic mirror (FF700-SDi01, Semrock, Rochester, NY, USA) placed at the turret box of the microscope and is focused into the sample with a 20 × 0.8 NA objective-lens (Plan-Apochromat 20x/0.8 NA, Carl Zeiss). The emitted fluorescence and SHG signals are collected by the same objective and firstly are filtered by a short pass filter (FF01-680/SP, Semrock) to ensure that no laser light is propagating to the detector. Then a band-pass filter (FF1-620/52, Semrock) or a band-pass filter (FF1-514/3, Semrock) follows, which allows passing the wavelengths in a range of 620 ± 26 nm or in a range of 514 ± 1.5 nm, respectively, before reaching a detector, based on a photomultiplier tube PMT (H9305-04, Hamamatsu, Hizuoka, Japan). The FF1-620/52 is used to detect the 2p-F from Biotracker 650 Red Nuclear Dye while the FF1-514/3 is used to detect the SHG from graphene.

Coordinated motion of the galvanometric mirrors and the detector for image acquisition is performed using custom-built Labview (National Instruments, Austin Texas, USA) software.

## Results & discussion

### Characterisation of exfoliated graphene

**Fig. 2:**
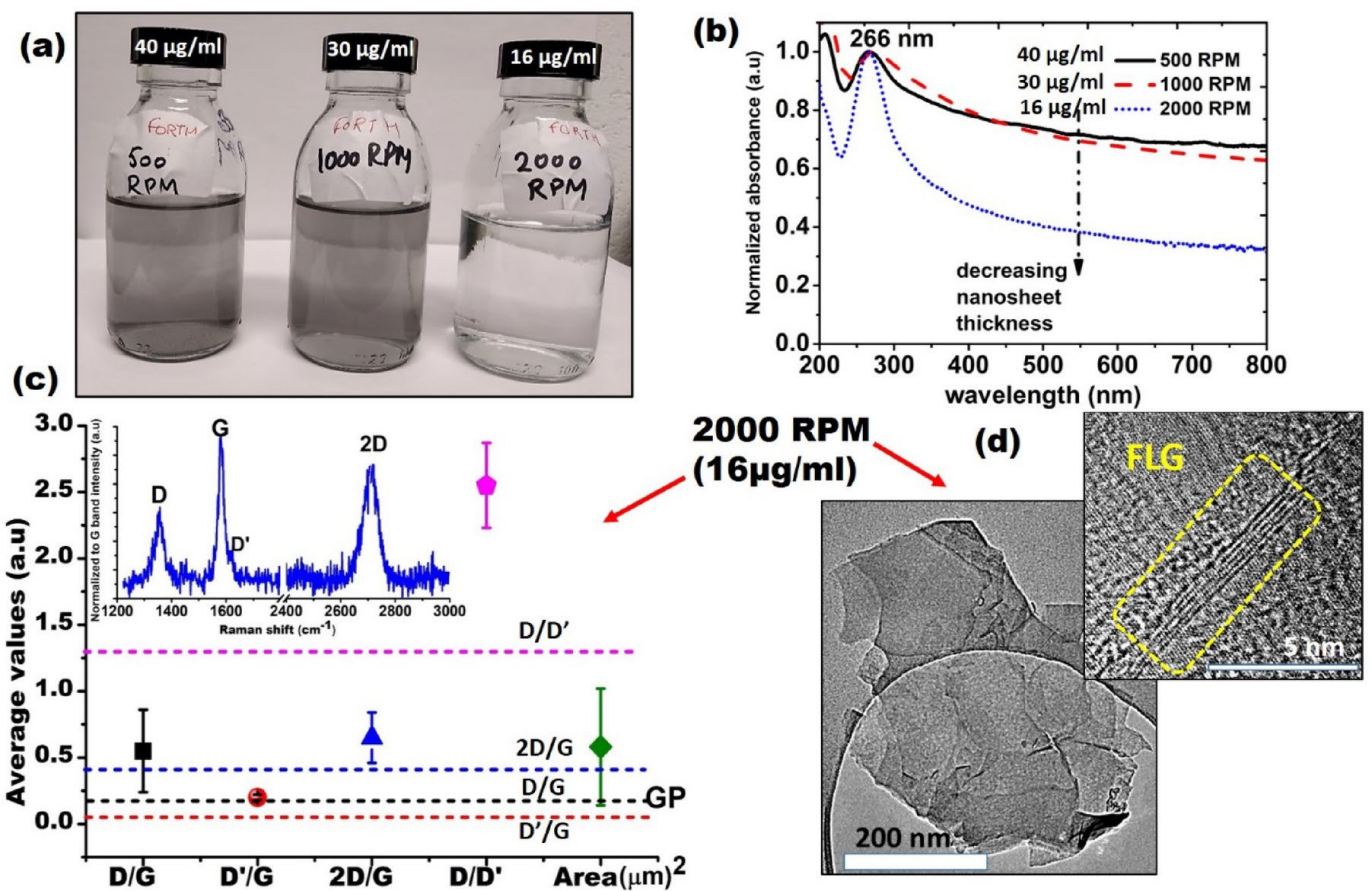
(**a**) Images of supernatants obtained after centrifugation at 500 (40 µg/ml), 1000 (30 µg/ml) and 2000 RPM (16 µg/ml); (**b**) Normalised UV-Vis absorption graph for the obtained supernatants; (**c**) Averaged intensity ratios of peaks, D/G (square), D′/G (sphere), 2D/G (triangle), D/D′ (pentagon) and area of TEM registered flakes (diamond) in 2000 RPM sample. The intensity ratio data for the original GP is indicated with dashed lines of the same colour for reference; inset shows a representative Raman spectrum of exfoliated flakes in 2000 RPM (16 µg/ml) sample, featuring D, G, D’ and 2D peaks; The error bars represent standard deviation of data; d) Typical TEM and HR-TEM image of exfoliated flakes in 2000 RPM (16 µg/ml) sample, dotted rectangle indicates FLG.

The appearance and microscopic features of the supernatants produced after ULPE, centrifuged at 500 (40 µg/ml), 1000 (30 µg/ml) and 2000 RPM (16 µg/ml) are shown in Fig. 2. It is evident from visual inspection of Fig. 2a that the concentrated supernatants obtained at 500 (40 µg/ml), and 1000 RPM (30 µg/ml) were dark-greyish transparent, indicating that the suspensions were enriched with thick multi-layer flakes and aggregates. In contrast, the supernatant obtained after 2000 RPM (16 µg/ml) was translucent, suggesting the presence of thin graphene flakes. UV-Vis spectroscopy was suitable to detect graphene-related absorption peaks in the 200–700 nm wavelength range. A typical absorption peak ~ 266 nm attributed to the π-π* transitions of the C = C sp^2^bonds^23^ was clearly seen, which confirms the presence of graphene flakes in each suspension (Fig. 2b). Variations in the slopes of normalised absorption curves were noted. Such variations are indicated with a dashed arrow in Fig. 2b. The downward arrow points to a tendency of thickness decrease of the nanosheets, which is in good agreement with previous studies^24^ and implies that the 2000 RPM (16 µg/ml) sample contained thin graphene flakes and was devoid of thick material. Based on the UV-Vis results described above, the 500 (40 µg/ml) and 1000 RPM (30 µg/ml) samples were excluded from subsequent Raman and HR-TEM studies. Raman spectroscopy was employed to evaluate the defects and thicknesses of exfoliated flakes qualitatively. Figure 2c shows relevant intensity ratio parameters such as D/G, D′/G, 2D/G, D/D′ derived from each recorded Raman spectrum for the 2000 RPM (16 µg/ml) sample. All the spectra were normalised to the intensity of the G-band. A representative Raman spectrum of FLG suspended in 2000 RPM (16 µg/ml) sample with prominent graphene signature bands D (1350 cm^−1^), G (1580 cm^−1^), D′ (1620 cm^−1^) and 2D (2700 cm^−1^)^25^ is shown in Fig. 2c. From the intensity ratio parameters, defect ratios D/G (0.55 ± 0.31), D′/G (0.2 ± 0.02) and D/D′ (2.55 ± 0.32) fall within the acceptable limits of typical solvent exfoliated graphene flakes^26^. At the same time, the 2D/G values (0.65 ± 0.19) clearly establish the FLG character, which is in accordance with the UV-Vis measurements (Fig. 2b). For morphological characterizations that included the quantification of size and number of graphene layers, conventional and high-resolution TEM (HR-TEM) were used. As shown in Fig. 2c, the average area of exfoliated flakes was 0.5 µm^2^. Figure 2d shows a representative HR-TEM image of graphene flakes exfoliated in 2000 RPM (16 µg/ml). The FLG character is indicated by a yellow rectangle. In this case the thickness is 5-layers with the average thickness ranging from 1.5 to 2 nm.

### In vitro cell viability

**Fig. 3:**
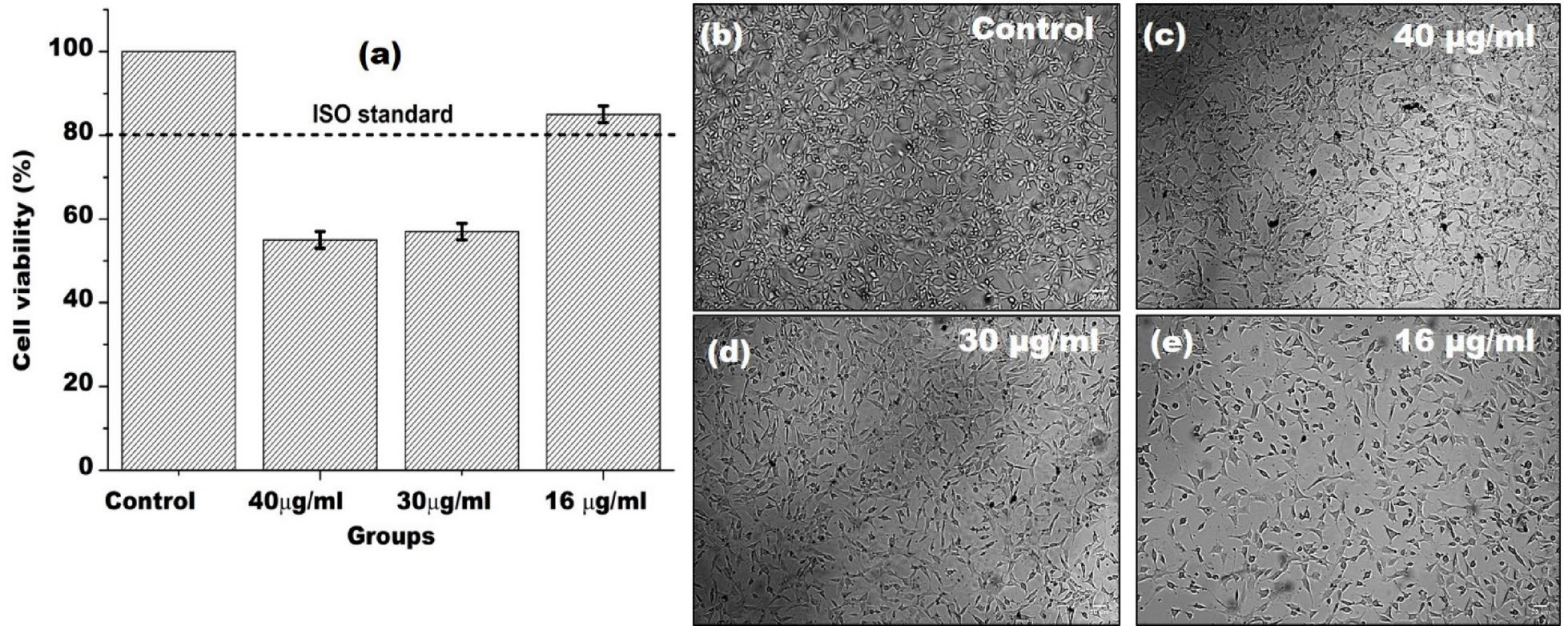
(**a**) Cell viability of mouse NIH 3 T3 fibroblast cells determined by the MTT assay after 24-h exposure to different concentration of FLGs (The bars indicate the mean ± SD deviation). Optical microscopic images of fibroblasts at 10× magnification (scale bar 20 μm). (**b**) Control group in DMEM medium, high glucose without FBS (c)-(e) Graphene samples in cell extracts prior to the addition of MTT.

According to the ISO 10993-5 standard, a substance can be considered non-cytotoxic if it permits cell viability to be higher than 80% after exposure for 24 h. It is evident from the data shown in Fig. 3a-e that FLG samples recovered after 500 (40 µg/ml) and 1000 RPM (30 µg/ml) can be deemed cytotoxic, possibly because of the variable graphene content i.e. thick flakes or aggregates. The MTT assays presented in Fig. 3a revealed that 500 RPM (40 µg/ml) graphene flakes reduced cell viability by about 45% in comparison to the control group, whereas the 1000 RPM group (30 µg/ml) decreased cell viability by about 42%. On the other hand, the 2000 RPM (16 µg/ml) group reduced cell viability by less than 20% in comparison to the control medium, thus adhering well to the ISO standard. The optical microscopic images (Fig. 3b-e) demonstrate that fibroblasts were decreased in number compared to the control, with maximum concentration of live cells. Thick aggregates were seen in concentrated samples i.e. 40 µg/ml and 30 µg/ml (Fig. 3c-d). Overall, our studies show that cell viability was proportionate to centrifugation speed and inversely proportional to the concentration of graphene flakes and their thicknesses. Hence, the higher the speed of centrifugation the smaller the thickness of the graphene flakes (or less aggregates) and consequently, the higher the viability of the cells.

### Cell morphology: live cell imaging with graphene

**Fig. 4:**
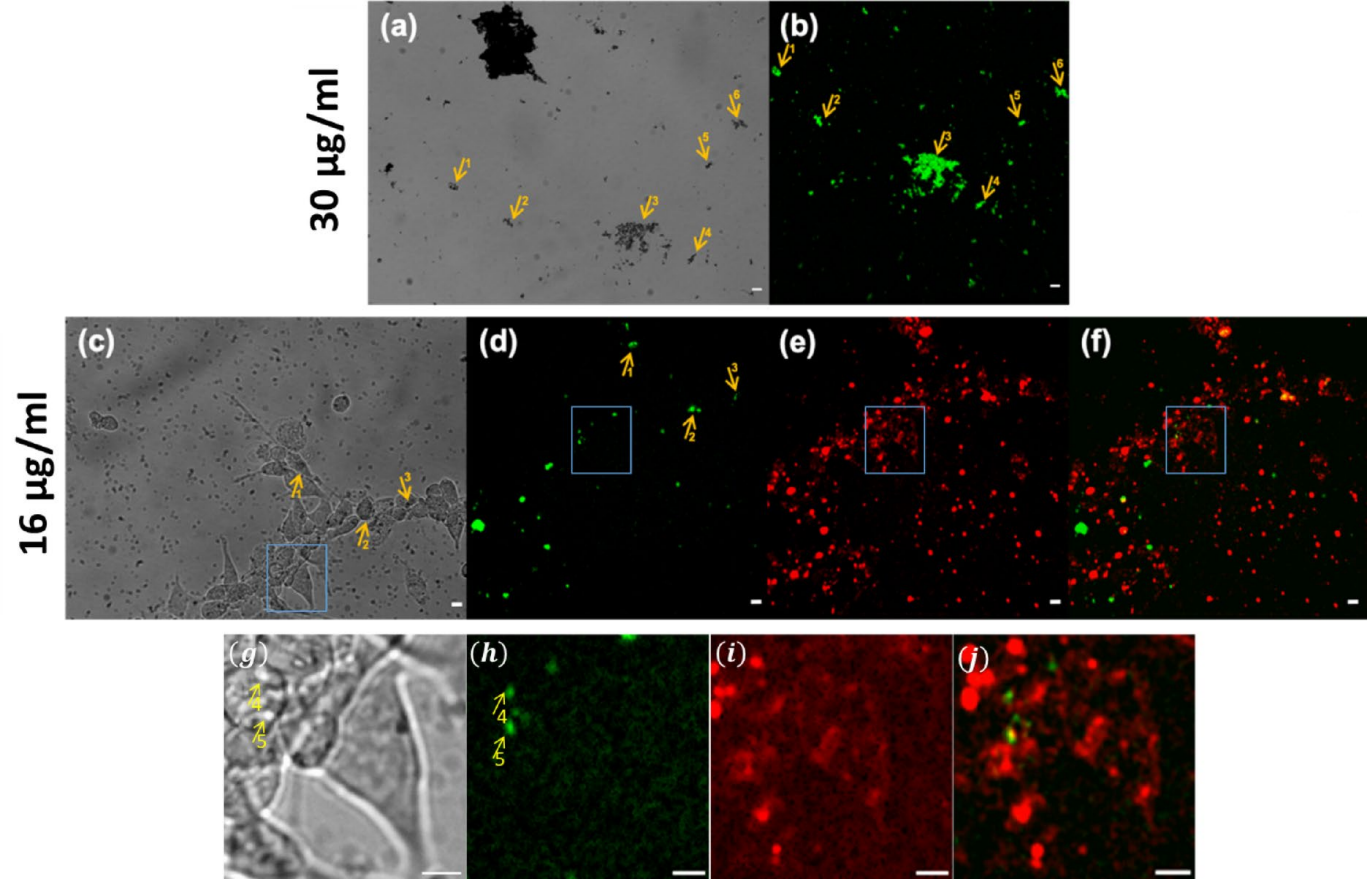
(**a)** Widefield Charge coupled device (CCD); (**b)** SHG images of pure graphene flakes (green), pointed with yellow arrows recorded for 30 µg/ml sample as a reference; **(c)** Wide field CCD images of live NIH 3 T3 cells in graphene solution (16 µg/ml); **(d)** Second Harmonic Generation **(**SHG) of graphene flakes at concentration 16 µg/ml in live cells; **(e)** 2p-F images of the nuclei of live cells in the graphene solution (16 µg/ml). Nuclei of live cells were labelled with Biotracker 650 Red Nuclear dye; (**f)** Combined images from SHG and 2p-F images. Cells are depicted by 2p-F imaging, while graphene flakes are depicted mainly by SHG imaging. 2p-F and SHG signals are represented by red and green, respectively; (**g)-(j)** CCD, SHG, 2p-F and merged SHG/2p-F signals seen in the rectangles of (**c)-(f)**, respectively. All scale bars show: 10 μm.

After quantification of the nanomaterial cytotoxicity in human cells in culture, we set out to examine live cells for changes in general morphology, detachment, cell lysis, and membrane integrity^27^. Gross alterations in membrane permeability, morphological, and physiological conditions can affect cell viability^5^. Figures 4a, b show wide-field CCD and SHG images of graphene flakes recorded as a reference for 30 µg/ml sample. Figures 4c-f, show real time live NIH 3 T3 cells in the presence of graphene flakes (pointed with yellow arrows in Fig. 4c) and SHG signals (Fig. 4e-f). As shown in Fig. 4d, label-free graphene flakes at a concentration of 16 µg/ml and cell nuclei could be observed using our custom-built multiphoton microscope. This was in agreement with the label-free imaging of pure graphene flakes performed prior to the experiments presented in Fig. 4b. Additionally, Fig. 4g-j present CCD, SHG, 2p-F and merged SHG/2p-F signals seen in the rectangles of 4c-f, respectively. Although centrosymmetric graphene monolayer was not expected to produce SHG signals^28^the few/multilayer graphene can produce SHG^29^. Live cell imaging provides dynamic information on cell morphology, interactions, and responses to the microenvironment details that endpoint viability assays cannot capture. Using our custom-built multiphoton microscope, we were able to simultaneously detect, in real-time, both the Biotracker 650 Red Nuclear Dye and the SHG signal from the label-free graphene flakes, providing evidence of safe, non-toxic cell-flake interactions.

### Cytotoxicity profile of FLG in human fibroblasts and HeLa cells

**Fig. 5:**
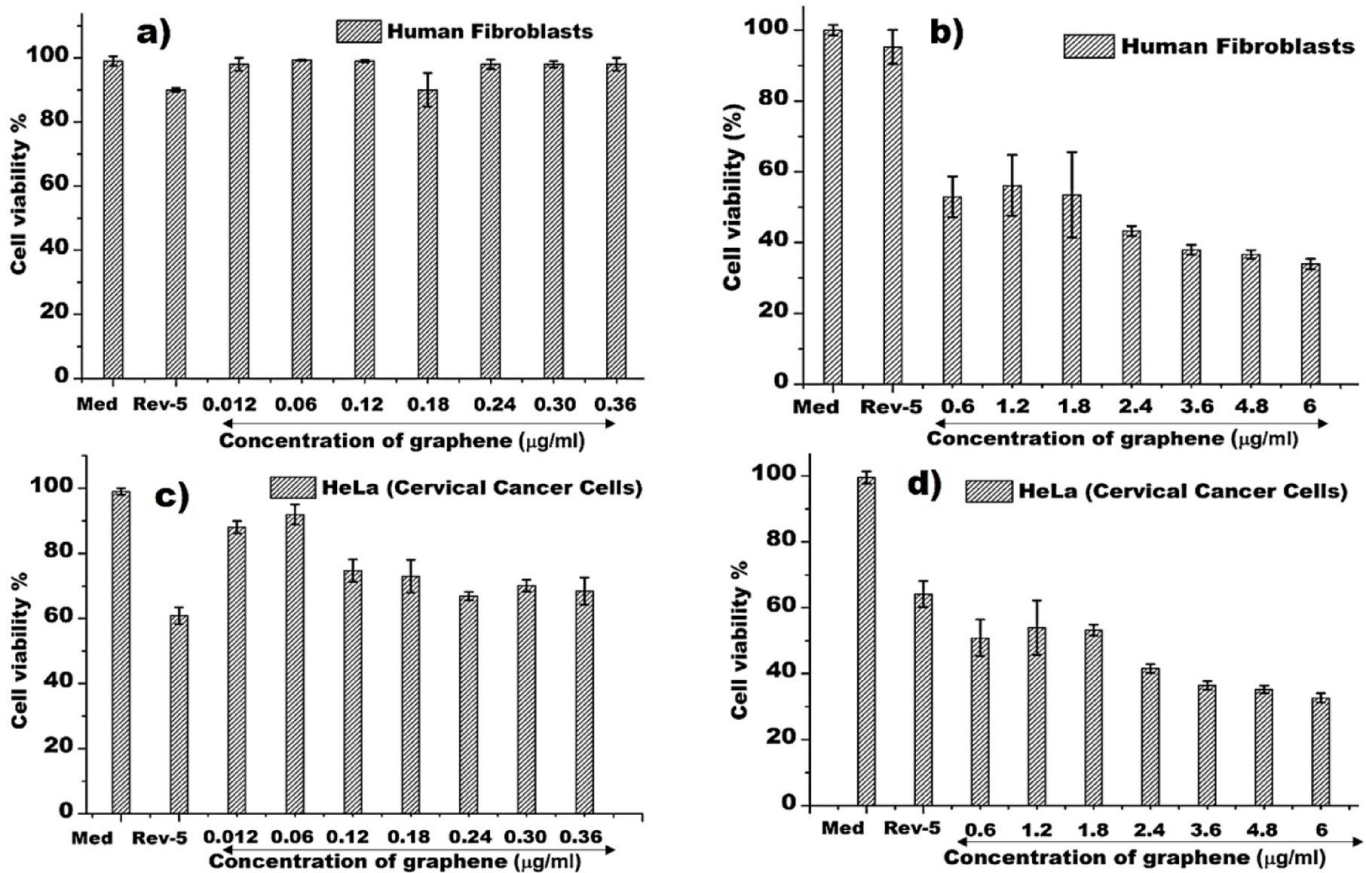
Graphene flakes dose-dependent cytotoxic profile, (**a**) cell viability (mean) of human fibroblasts treated with low concentration (0.012–0.36 µg/ml) and (**b**) high-concentration (0.6–6 µg/ml) of FLG. (**c**) cell viability (mean) of HeLa cells treated with low concentration (0.012–0.36 µg/ml) and (**d**) high-concentration (0.6–6 µg/ml) of FLG for 72 h. Rev-5 represents Reversine at 5 µM concentration (9.8 µg/ml). The error bars represent standard deviation of three measurements.

The FLG cytotoxicity profile observed in mouse fibroblasts utilizing aqueous graphene (Figs. 3 and 4), prompted us to investigate the cytotoxicity of FLG towards human fibroblasts and cancer cells. Figure 5a–d depicts the dose-dependent cytotoxic profile of FLG (exfoliated in PBS) in human fibroblasts and HeLa cells. The data in Fig. 5a demonstrated a very low cytotoxicity (> 90% cell survival) of the FLG in human fibroblasts when used at 0.012–0.36 µg/ml concentration. For comparison, cell toxicity experiments of fibroblasts exposed to the strong antimitotic drug Reversine were carried out and a 15% decrease of cell viability observed (Fig. 5a, b). This investigation in human fibroblasts correlated well with > 80% mouse fibroblast cell survival observed in FLG extracts (16 µg/ml), as previously mentioned in Sect. 3.2 and shown in Fig. 3a. Employing FLG at higher concentrations (0.6–6 µg/ml) was highly cytotoxic to human fibroblasts, resulting in 50–60% cell death (Fig. 5b). Taken together, these findings indicate that our graphene is non-cytotoxic to healthy human fibroblast cells at low concentrations. Interestingly, graphene in water and PBS showed lower cytotoxicity (~ 80% and > 90% cell survival, respectively), as shown in Figs. 3a and 5a, respectively, suggesting a potential usage of FLG as a bio friendly drug carrier.

Importantly, FLG flakes killed ~ 30% of HeLa cells when used at lower concentrations (0.12–0.36 µg/ml range; Fig. 5c). This level of cytotoxicity to Hela cells was comparable to that caused by the antimitotic drug Reversine used at 5 µM (9.8 µg/ml), suggesting graphene at these concentrations could potentially be used to sensitise cancer cells before chemotherapy, radiotherapy or both. The fact that different types of nanomaterials can sensitise cancer cells of different tissue origin, including chitosan-based nanoparticles^30,31^ adds support to this notion. Furthermore, FLG at higher concentrations (0.6–6 µg/ml) effectively kill HeLa cells, resulting in 50–60% cell death (Fig. 5d). However, at such high concentrations, graphene flakes also exhibited a high cytotoxic effect in non-transformed, human fibroblasts. Future work will aim to define the molecular mechanism(s) that enable the selective killing of cancer cells and to establish the extent of cancer cell sensitization by FLG in cancer cells of different tissue origins and poor prognosis such as pancreatic, prostate and lung cancer.

## Conclusions

This study reports the preparation and characterisation of eco-friendly FLG and its effects on cell toxicity. This includes tests on mouse fibroblasts NIH 3 T3, human fibroblasts and HeLa cancer cells exposed to FLG in aqueous solutions, biological buffers and cell culture media. We demonstrated that our graphene product is non-toxic to fibroblasts but highly cytotoxic to cervix cancer cells, achieving a killing rate of ~ 30% at low micromolar concentrations (0.012–0.36 µg/ml). The effect of FLG on the latter cell line was comparable to the cytotoxicity of the antimitotic drug Reversine used at a similar concentration. Our study also demonstrated the bio-friendly nature of our graphene product and its potential use for cancer cell sensitisation without affecting human cells in addition to its use as a drug nanocarrier. We anticipate that FLG dispersions prepared in a bio-friendly system will open up new opportunities for the use of graphene in biotechnology and pharmacology. Future research work will aim to investigate the underlying molecular mechanisms responsible for cervix cancer cell death induced by graphene and the relative cytotoxicity of FLG on cancer cells from different tissue origin and poor prognosis.

## Data Availability

The data that supports the findings of this study are available upon request from the corresponding authors. Correspondence and requests for materials should be addressed to AK and IT.
